# Prevalence of Pregnancy Associated Malaria in India

**DOI:** 10.3389/fgwh.2022.832880

**Published:** 2022-05-26

**Authors:** Khushi Jain, Palak Gupta, Ashutosh Balodhi, Farah Deeba, Nasir Salam

**Affiliations:** ^1^Department of Microbiology, Central University of Punjab, Bathinda, India; ^2^Centre for Interdisciplinary Research in Basic Sciences, Jamia Millia Islamia, New Delhi, India

**Keywords:** malaria, pregnancy, prevalence, still birth, abortion

## Abstract

Malaria in pregnancy is a major public health concern. It results in impaired maternal health and adversely effects fetal and perinatal outcomes. The present systematic review was conducted to assess the prevalence, distribution and adverse pregnancy outcomes in malaria infected females in India. A comprehensive search and review of PubMed and Web of Science based on PRISMA guidelines was carried out to find articles reporting prevalence of malaria in pregnant women from India. Data from 16 studies were analyzed and prevalence of malaria among pregnant women in India was found to be 11.4 % (95 % CI: 7.3, 16.3). Prevalence of malaria among asymptomatic and symptomatic pregnant women was found to be 10.62% (95% CI: 6.05, 16.23) and 13.13% (95% CI: 7.2, 20.52), respectively. *P. falciparum* and *P. vivax* were both reported with in the same population. The geospatial distribution of malaria in pregnancy spanned over nine very populous states of India. The review also reported severe maternal and perinatal outcomes. Given the seriousness of malaria in pregnant women and its effects on the fetus and new-born, a stringent district wise guideline for early detection and prophylaxis in regions identified in this review will help in its better control.

## Introduction

Malaria continues to be a serious public health concern with 229 million cases and 409,000 deaths reported in the year 2019. Nearly 6.3 million cases were reported from Southeast Asia region out of which majority were present in India^1^. In the last two decades, there has been a sustained and focussed effort to reduce malaria infection by health agencies with some degree of success, even so areas of high burden among the vulnerable populations continues to be a problem (1).

Pregnant women are one such group which shows increased risk of malaria in endemic areas with potentially life-threatening consequences for mother, fetus, and the neonate. Malarial infection during pregnancy is associated with high risk of anemia, miscarriages, preterm deliveries, low birth weight, congenital malaria, and deaths of infants (2). Several studies have been carried out that have estimated the burden of malaria in pregnant women from sub-Saharan Africa (3–6). However, there is lack of comprehensive data from India, regarding pregnancy associated malaria and adverse pregnancy outcomes.

Though there is distinct lack of statistical data published for malaria in pregnancy from India, yet there are several studies that analyse its implications. A study published from Gujarat, West India, from the period of 1987–1988, showed that pregnant women are more prone to malaria infection than non-pregnant women (7). In another study from Odisha, east India, it was found that neonates are more prone to infection and parasitaemic infection are more common in primigravidae than multigravida women (8). Prevalence of malaria in pregnancy in health care centers from Madhya Pradesh was found to range between 6.4 and 55% approximately (9).

Even a cursory analysis of the prevalence of malaria in pregnant women indicates that in endemic regions pregnant women are more prone to malaria infection as compared to non-pregnant women, resulting in severe consequences for the health and wellbeing of the mother, fetus and infant.

Many individual studies from India are available looking at sub-national estimates of malaria in different parts of the country. A comprehensive analysis of the burden of malaria during pregnancy from India is urgently required. Such studies have the potential to guide targeted public health efforts toward the affected population. The present study is a systematic review and meta-analysis of published literature to estimate the proportion of malaria in pregnant women and pregnancy outcomes associated with malaria infection from India.

## Methodology

### Search Strategy

PRISMA (Preferred reporting items for systematic reviews and meta-analysis) guidelines was the basis of our review to extract all the relevant publications reporting the prevalence of malaria in pregnancy from India. We systematically searched PubMed and Web of Science focusing on the time span between the years 2000 and 2020 with the help of the following keywords—“Malaria or plasmodium” AND “pregnancy or pregnant” AND “India.” The searches were transferred to open-source citation management software Zotero and further assessed for eligibility and quality.

### Eligibility Criteria

Preliminary screening was carried out by reviewing the title and abstract of the studies. Full text of potentially relevant articles was further analyzed for the availability of prevalence data for malaria during pregnancy. In the final draft all the case reports, retrospective analysis, cross-sectional studies with availability of full-text articles and reporting prevalence of malaria in pregnancy were included. Abstracts, reviews, conference proceedings, studies reporting malaria in non-pregnant/non-female subjects etc. were all excluded. The quality of articles was assessed using Joana Brigg's Institute (JBI) critical appraisal checklist for simple prevalence and studies scoring ≥5 was included in the final meta-analysis (10).

### Data Extraction

The following information was extracted from all included publications: First author, nature of study, date and location of study, sample size, age, gender, type of diagnostic test performed, maternal health, pregnancy outcomes, and the *plasmodium* species detected. Data was then compiled in a tabulated form and it was later cross verified by various authors to avoid discrepancy.

### Data Analysis

Data from eligible primary studies were used for calculating pooled prevalence of malaria in pregnant women in India. MedCalc version 20.014 was used for statistical analysis. Pooled prevalence was calculated with 95% confidence interval and data was displayed with both random effects model and fixed effects model. Cochran's Q test and *I*^2^ statistics were used to calculate the variance between studies and heterogeneity in estimates. Funnel plot was generated to evaluate publication bias.

### Geospatial Map

The data extracted only from cross sectional studies was used to create a map using the free online map maker Datawrapper (http://www.datawrapper.de). The data was entered in the tabulated form in Datawrapper tool based on the geographical location from where it was reported.

## Results

**Figure 1:**
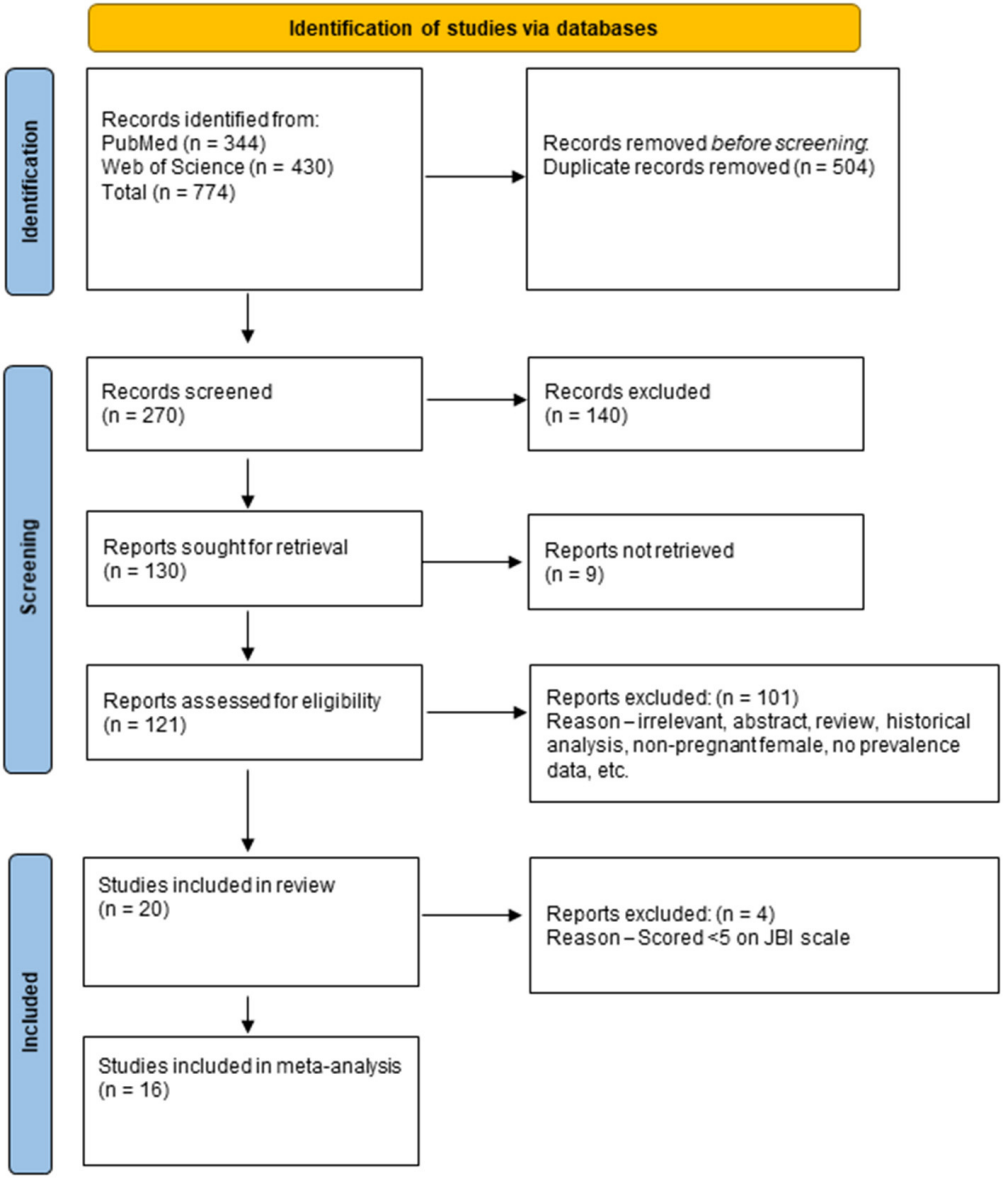
PRISMA flow chart for study selection process.

Our initial systematic search of PubMed and Web of Science resulted in 774 research articles pertaining to malaria in pregnancy reported during years 2000–2020 from India. After removal of duplicates, 270 unique articles were identified and they were reviewed for title and abstract. Out of these 130 were considered for full text review (Figure 1).

Finally, twenty articles were ultimately found to fulfill the eligibility criteria and were included in the final systematic review (11–30). Among these 20 articles, 11 were cross sectional studies, three were retrospective analysis, 5 were hospital based observational studies and the remaining one was a case series (Table 1).

**Table 1:** Overview of the published reports considered for final analysis.

**Characteristic**	***n*** **(%)**
No. of studies	20 (100)
**Studied population**	
Pregnant women	20 (100)
**Parasite species reported**	
*Plasmodium falciparum*	1 (5)
*Plasmodium vivax*	1 (5)
*P. falciparum* and *P. vivax*	6 (30)
*P. falciparum, P. vivax*, and Mixed infection	6 (30)
Not defined	6 (30)
**Examination method**	
Microscopy	5 (25)
RDT	2 (10)
Microscopy, RDT^*^	7 (35)
Microscopy, PCR^**^	1 (5)
Microscopy, ELISA^***^	1 (5)
Microscopy, RDT, Placental histology, PCR	1 (5)
Not defined	3 (15)

* *RDT, rapid diagnostic test*.

** *PCR, polymerase chain reaction*.

*** *ELISA, Enzyme linked sorbet assay*.

These studies were carried out in pregnant females within the age group ranging from 18 to 45 years. Many of the studies were reported primarily from the states of Madhya Pradesh, Chhattisgarh, and Jharkhand. Quality analysis using JBI criteria resulted in 16 studies scoring ≥ 5. Among these 16 studies, 11 studies reported malaria in asymptomatic pregnant women and five studies reported malaria in symptomatic women. The symptom commonly associated with malaria was fever. These 16 studies were finally included in the pooled prevalence analysis.

### Characteristics of Included Studies

Our review focused on maternal characteristics and all the possible outcomes of malaria in pregnancy including low birth weight, preterm delivery, abortions and stillbirth. Including all the 20 articles, the total sample size of pregnant women tested were 24,052, out of which 416 reported fevers, 754 were admitted to Intensive care unit (ICU) and 120 were deceased (Table 2).

**Table 2:** Overview of the extracted data considered for the systematic review.

**S. No**	**References**	**Year**	**Location**	**Study design**	**Sample size**	**Age**	**Diagnostic test**	**Positive samples**	**% Prevalence**	**Parasite sp**.
1.	Ahmed et al. (11)	2006–2007	Madhya Pradesh	Cross sectional Asymptomatic	506	20–29 yrs.	Microscopy, RDT, placental histology, PCR	38	7.5	PF–28 PV–11
2.	Singh et al. (12)	1997–1998	Madhya Pradesh	Cross sectional Asymptomatic	274	ND	Microscopy	151	55.1	PF–133 PV–18
3.	Singh et al. (13)	2002–2004	Madhya Pradesh	Cross sectional Asymptomatic	799	18–45 yrs.	Microscopy, RDT	86	10.8	ND
4.	Bardaji et al. (14)	2008–2011	Rajasthan	Cross sectional Asymptomatic	1,982	Mean−23.1 yrs.	Microscopy, PCR	26	1.3	PF–1 PV–25
5.	Hamer et al. (15)	2006–2007	Jharkhand	Cross sectional Asymptomatic	3,104	20–34 yrs.	Microscopy, RDT	55	1.7	PF–32 PV–18 Mixed–5
6.	Kupfer et al. (16)	2012–2015	Gumla, Simdega	Cross sectional asymptomatic	6,868	20–30 yrs.	Microscopy, RDT	111	1.6	PF–91 PV–11 Mixed–9
7.	Singh et al. (17)	2014–2015	Hazaribagh	Cross sectional asymptomatic	534	18–38 yrs.	Microscopy, RDT	50	9.4	ND
8.	Sohail et al. (18)	2012–2013	Hazaribagh	Cross sectional Asymptomatic	2,141	18–37 yrs.	Microscopy, RDT	105	5	PF–5 PV–91 Mixed–9
9.	Singh et al. (19)	2007–2008	Bastar Rajnandgaon	Cross sectional Asymptomatic	3,721	20–34 yrs.	Microscopy, RDT	55	1.5	PF–42 PV–11 Mixed–2
10.	Corrêa et al. (20)	2015	AP Chhattisgarh Telangana	Cross sectional Asymptomatic	575	Median age–26 yrs.	RDT	165	28.7	PF–106 PV–1 Mixed–58
11.	Singh et al. (21)	DoP−2014	Rewa	Observational Asymptomatic	203	ND	Microscopy	72	35.4	PF–12 PV–60
12.	Chauhan et al. (22)	2007–2011	Bastar	Retrospective Symptomatic	120 Deceased women	18–42 yrs.	ND	15	12.5	ND
13.	Guin et al. (23)	2008–2009	Jabalpur	Cross sectional Symptomatic	500	24.5 ± 2.6 yrs.	Microscopy, RDT	37	7.4	PF–26 PV–5 Mixed–6
14.	Qureshi et al. (24)	2012	AP Chhattisgarh	Retrospective Symptomatic	1,222	ND	RDT	131	10.7	ND
15.	Munnur et al. (25)	1992–2001	Mumbai	Retrospective Symptomatic	754	25.4 ± 4.6 yrs.	ND	75	10	ND
16.	Chawla and Manu (26)	DoP−2007	Mumbai	Observational Symptomatic	416	ND	Microscopy	27	6.5	PF–13 PV–8
17.	Bhadade et al. (27)	2009–2010	Mumbai	Observational Symptomatic	122	21–30 yrs.	ND	19	15.6	ND
18.	Datta et al. (28)	2014–2015	Kolkata	Observational Symptomatic	183	17–35 yrs.	Microscopy, RDT	64	35	PF–15 PV–49
19.	Aleyamma (29)	2006	Vellore	Case series Symptomatic	3	30, 26, 22 yrs.	Microscopy	All	NA	PF
20.	Nayak et al. (30)	2009	Bikaner	Observational Symptomatic	25	ND	Microscopy	All	NA	PV–25

*ND, Not defined; DoP, Date of publication; PF, Plasmodium falciparum; PV, Plasmodium vivax; RDT, Rapid Diagnostic Test; PCR, Polymerase chain reaction; ICU, Intensive Care Unit; ELISA, Enzyme Linked Immunosorbent Assay; NA, Not applicable*.

**Figure 2:**
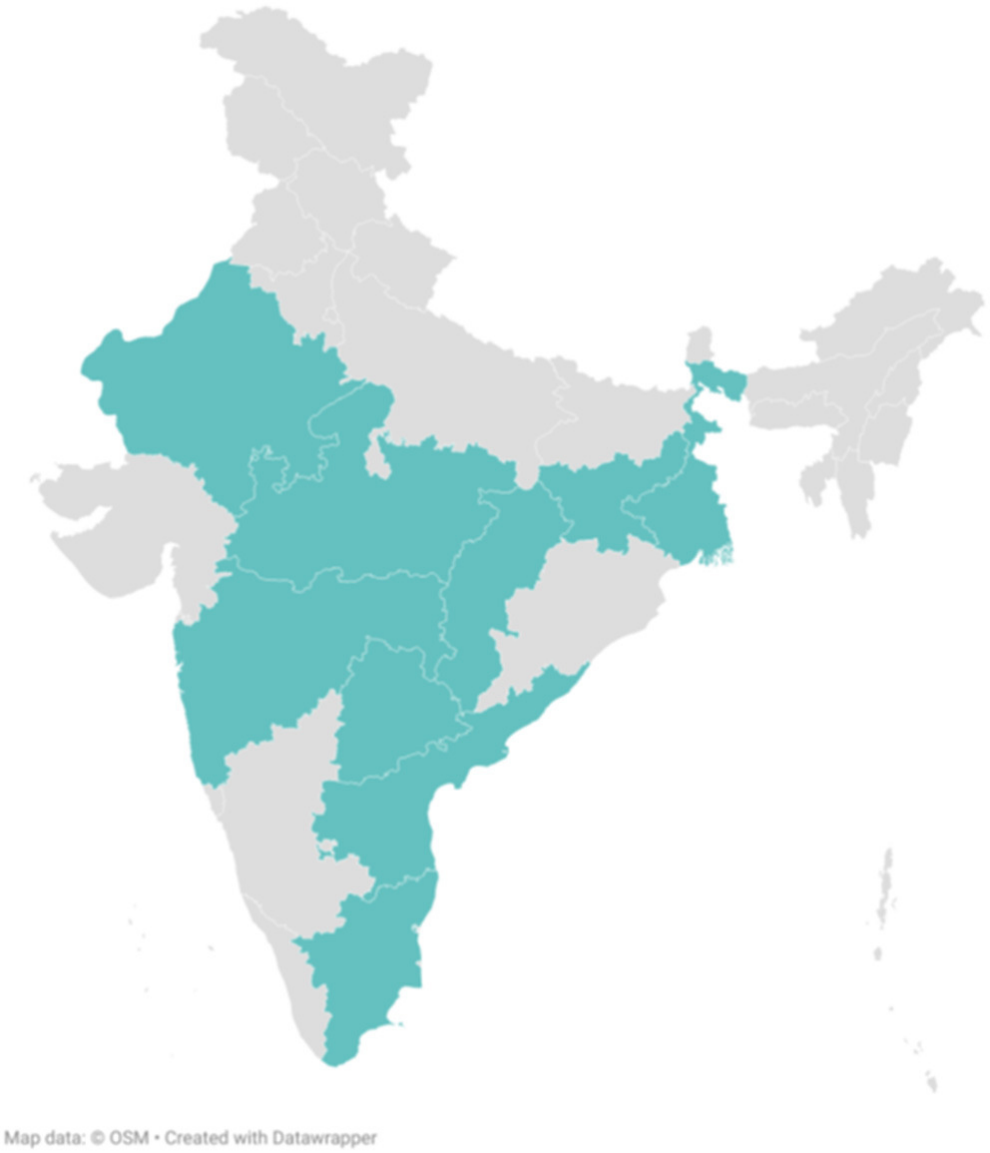
States from where studies were reported.

All the samples were tested for the two most prevalent species of malaria, *P. falciparum* and *P. vivax* either through microscopy, which was found to be most favored technique, because it is the most consistent and definitive way to identify the parasite. Other diagnostic methods were Rapid Diagnostic Test (RDT), Polymerase Chain Reaction (PCR) and placental histology. Some samples were also found to have mixed infection with both *P. falciparum* and *P. vivax*. Studies were reported from 9 out of 28 states and 18 districts of India, including Madhya Pradesh, Tamil Nadu, Rajasthan, Maharashtra, West Bengal, Jharkhand, Chhattisgarh, Andhra Pradesh, and Telangana (Figure 2).

Major characteristics reported in pregnant women with malaria was anemia, preeclampsia, thrombocytopenia, and pathological placenta (Table 3). Many studies reported, stillbirths, abortions, intrauterine deaths, preterm births, low birth weight, low Apgar score, and perinatal mortality associated with malaria in pregnancy (Table 4).

**Table 3:** Effect of malaria on pregnant women.

**S. No**	**References**	**Malaria positive patients**	**Maternal health**			
			**Anemia**	**Thrombocytopenia**	**Preeclampsia**	**Mortality**
1.	Ahmed et al. (11)	38	20 (Hb lower by 1.4 g/dl)	-	-	-
2.	Guin et al. (23)	37	All Peripheral smear positive–Mean Hb level–7.18 ± 2.31 Placental smear positive–Mean Hb level–5.6 ± 2.3 Smear negative–Mean Hb level–8.46 ± 1.60	-	12	9
3.	Singh et al. (12)	151	All	–	–	–
4.	Nayak et al. (30)	25	15 (Hb <5 g%)	14 (Platelet count <100,000)	-	-
5.	Bhadade et al. (27)	19	–	–	–	2
6.	Datta et al. (28)	64	54	–	–	–
7.	Aleyamma et al. (29)	3	–	–	–	1

**Table 4:** Effect of malaria on pregnancy outcome.

**S. No**	**References**	**Malaria positive patients**	**Pregnancy outcomes/Neonatal health**						
			**Mean gestational stage**	**Preterm labor**	**Abortion**	**Still birth**	**Low birth weight**	**Apgar score**	**Mortality**
1.	Ahmed et al. (11)	38	33 (Lower by 0.3–0.8 weeks)	-	-	4	16 (lower by 400 g)	-	-
2.	Guin et al. (12)	37	-	18	-	2	All Peripheral smear positive–Mean birth weight–2.21 ± 0.44 Placental smear positive– Mean birth weight–2.07 ± 0.29 Smear negative–2.36 ± 0.25	9 (Apgar score <5)	Perinatal mortality–14
3.	Singh et al. (13)	151	-	-	6	5	125 Malaria positive patients–Mean birth weight–2.25 kg Malaria negative patients– Mean birth weight–2.40 kg	-	Perinatal mortality–4
4.	Singh et al. (14)	Mandla–56 Maihar–30	-	-	-	-	All Mandla With placental malaria–Mean birth weight–2.19 ± 0.76 kg Without Placental malaria–Mean birth weight–2.37 ± 0.31 kg Maihar With placental malaria–Mean birth weight–2.47 ± 0.44 kg Without placental malaria–Mean birth weight–2.60 ± 0.44 kg	-	-
5.	Hamer et al. (16)	55	-	2	-	2	4	-	-
6.	Nayak et al. (26)	25	-	8	2	2	20 (Birth weight <2.5 kg)	-	Intrauterine death–2
7.	Chawla and Manu (27)	27	-	4	3	1	4 (Birth weight <2.5 kg)	-	Intrauterine death–1
8.	Datta et al. (29)	64	-	32	-	-	51	47 (Apgar score <7)	Perinatal mortality–3
9.	Aleyamma et al. (30)	3	-	-	-	-	-	-	Intrauterine death–3

### Prevalence of Malaria in Pregnant Women

**Figure 3:**
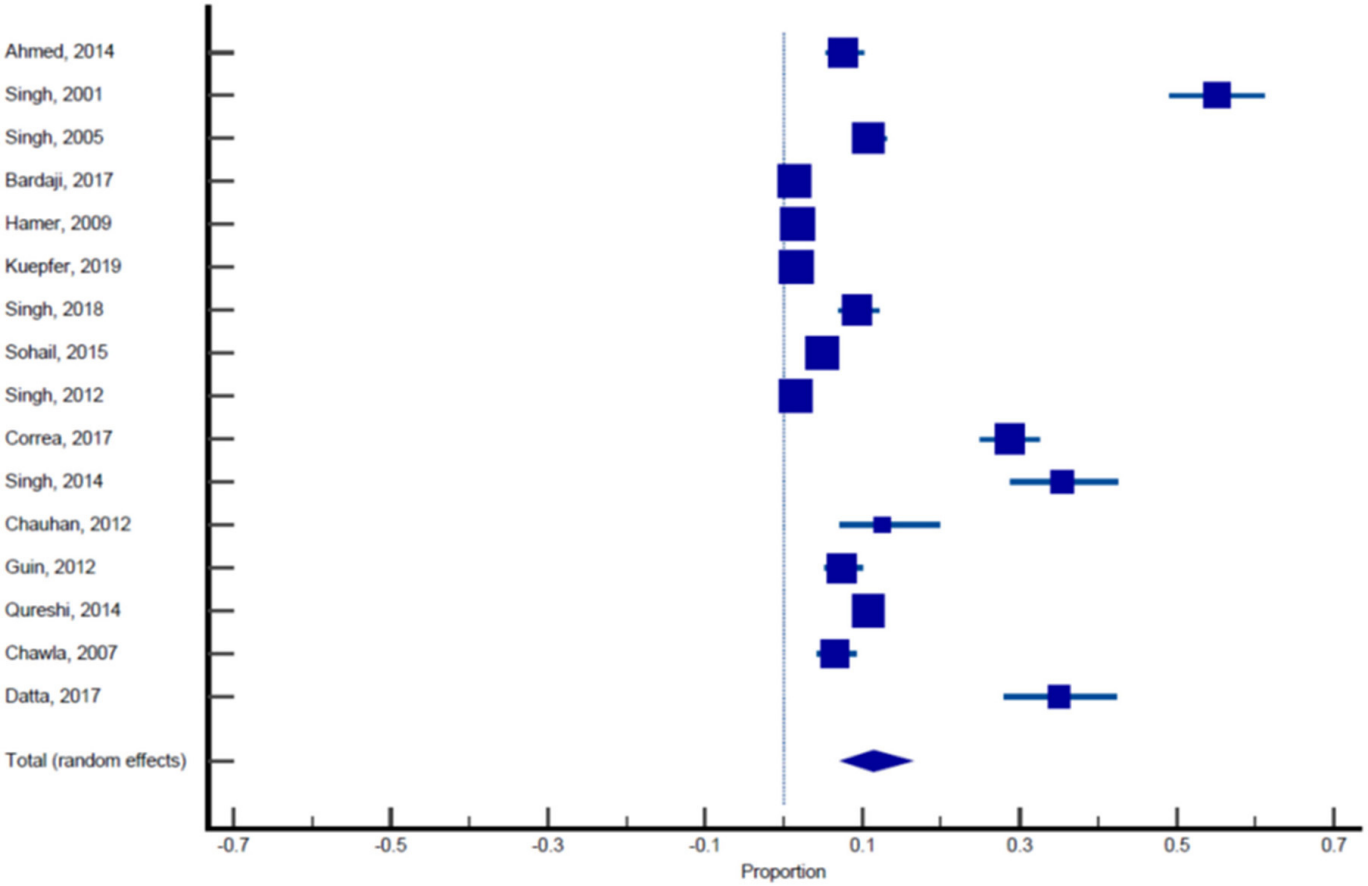
Forest plot showing prevalence of malaria in pregnant females, the blue diamond indicates the pooled prevalence and the horizontal line shows the 95% confidence interval.

**Figure 4:**
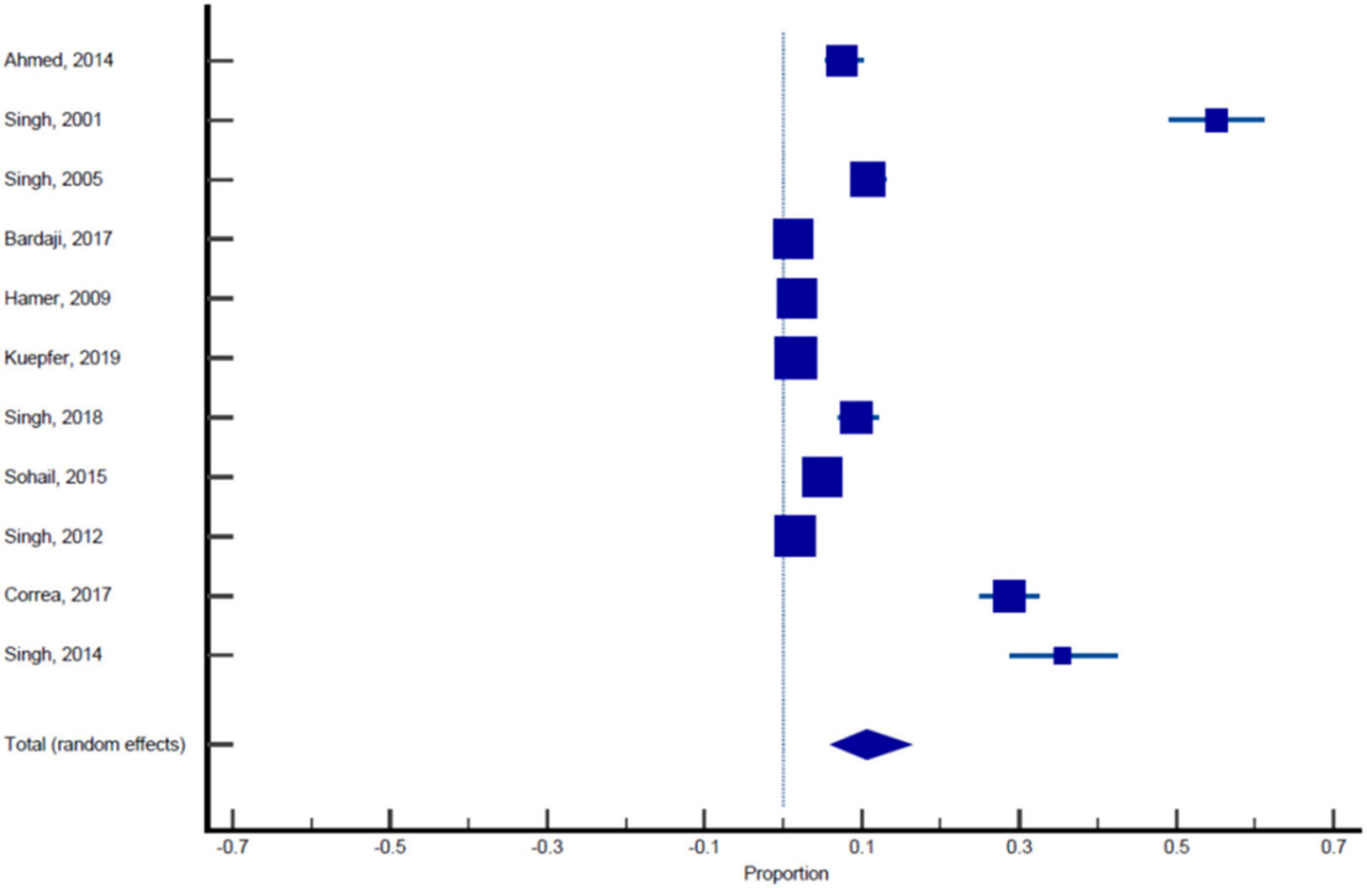
Forest plot showing prevalence of malaria in asymptomatic pregnant females, the blue diamond indicates the pooled prevalence and the horizontal line shows the 95% confidence interval.

**Figure 5:**
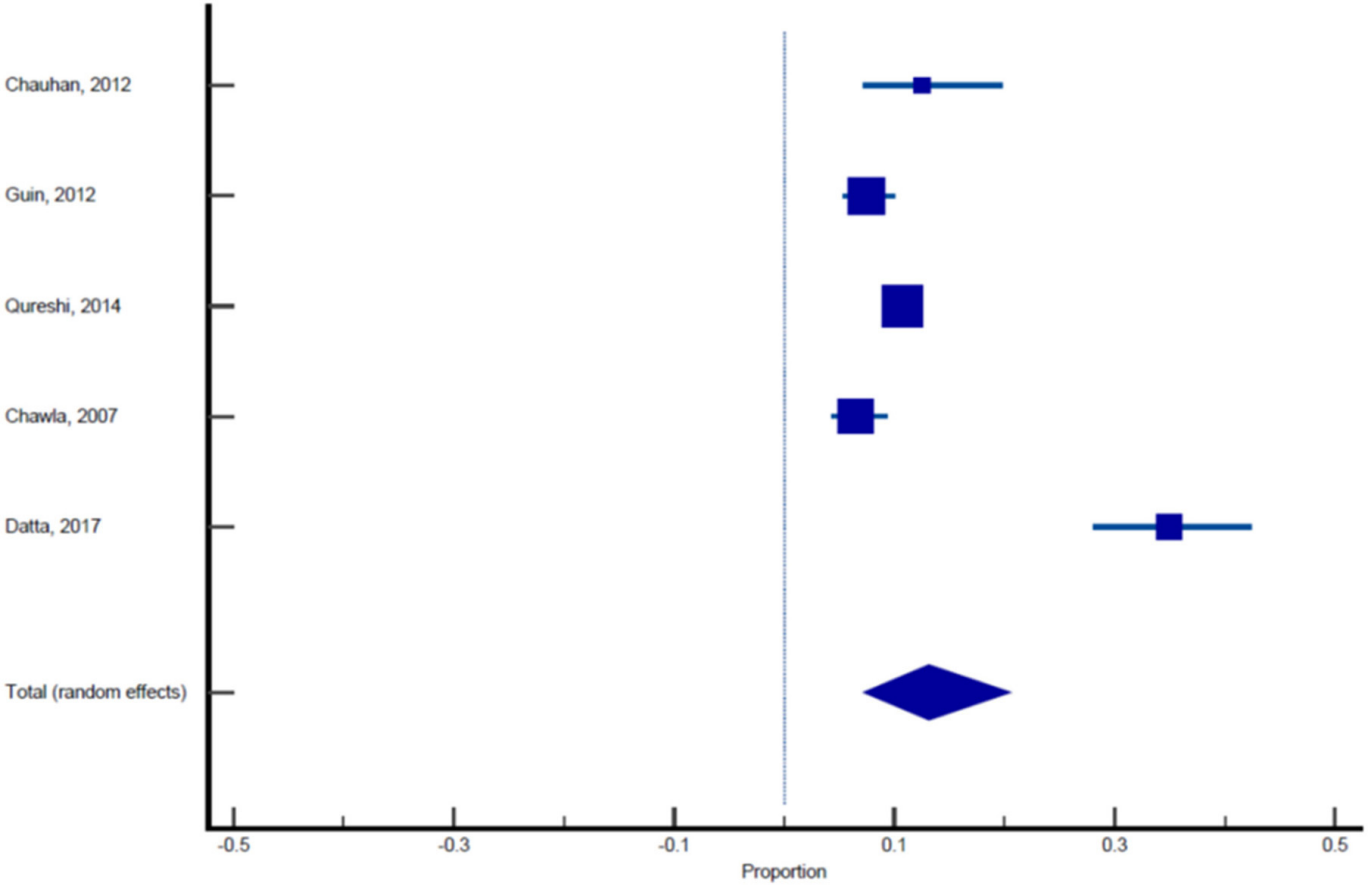
Forest plot showing prevalence of malaria in symptomatic pregnant females, the blue diamond indicates the pooled prevalence and the horizontal line shows the 95% confidence interval.

Sixteen published studies were included in the final meta-analysis and all these studies were used to estimate the pooled prevalence of malaria among pregnant women. Using the random effect analysis, the pooled prevalence of malaria among pregnant women in India was 11.4% (95% CI: 7.3, 16.3; Table 5, Figure 3). Subgroup analysis showed that the prevalence of malaria among asymptomatic and symptomatic pregnant women was 10.62% (95% CI: 6.05, 16.23; Table 6, Figure 4) and 13.13% (95% CI: 7.2, 20.52), respectively (Table 7, Figure 5).

**Table 5:** Pooled prevalence analysis of malaria in pregnant women across 16 studies.

**References**	**Sample size**	**Proportion (%)**	**95% CI**	**Weight (%)**	
				**Fixed**	**Random**
Ahmed et al. (11)	506	7.510	5.369–10.162	2.19	6.27
Singh et al. (12)	274	55.109	49.010–61.098	1.19	6.14
Singh et al. (13)	799	10.763	8.700–13.122	3.45	6.32
Bardaji et al. (14)	1,982	1.312	0.859–1.916	8.56	6.38
Hamer et al. (15)	3,104	1.772	1.338–2.300	13.40	6.39
Kuepfer et al. (16)	6,868	1.616	1.331–1.943	29.65	6.41
Singh et al. (17)	534	9.363	7.030–12.158	2.31	6.27
Sohail et al. (18)	2,141	4.904	4.028–5.906	9.25	6.38
Singh et al. (19)	3,721	1.478	1.115–1.920	16.07	6.40
Corrêa et al. (20)	575	28.696	25.029–32.583	2.49	6.28
Singh et al. (21)	203	35.468	28.897–42.471	0.88	6.05
Chauhan et al. (22)	120	12.500	7.168–19.778	0.52	5.83
Guin et al. (23)	500	7.400	5.264–10.056	2.16	6.26
Qureshi et al. (24)	1,222	10.720	9.041–12.591	5.28	6.35
Chawla and Manu (26)	416	6.490	4.320–9.303	1.80	6.23
Datta et al. (28)	183	34.973	28.085–42.356	0.79	6.02
Total (fixed effects)	23,148	3.789	3.547–4.043	100.00	100.00
Total (random effects)	23,148	11.398	7.294–16.277	100.00	100.00
**Test for heterogeneity**					
Q	1,612.7437				
DF	15				
Significance level	*P* < 0.0001				
*I*^2^ (inconsistency)	99.07%				
95% CI for *I*^2^	98.89–99.22				
**Publication bias**					
Egger's test					
Intercept	16.0846				
95% CI	9.8009–22.3683				
Significance level	*P* = 0.0001				
Begg's test					
Kendall's Tau	0.4167				
Significance level	*P* = 0.0244				

**Table 6:** Pooled prevalence analysis of malaria in asymptomatic pregnant women.

**References**	**Sample size**	**Proportion (%)**	**95% CI**	**Weight (%)**	
				**Fixed**	**Random**
Ahmed et al. (11)	506	7.510	5.369–10.162	2.45	9.04
Singh et al. (12)	274	55.109	49.010–61.098	1.33	8.86
Singh et al. (13)	799	10.763	8.700–13.122	3.86	9.12
Bardaji et al. (14)	1,982	1.312	0.859–1.916	9.57	9.21
Hamer et al. (15)	3,104	1.772	1.338–2.300	14.99	9.23
Kuepfer et al. (16)	6,868	1.616	1.331–1.943	33.15	9.25
Singh et al. (17)	534	9.363	7.030–12.158	2.58	9.05
Sohail et al. (18)	2,141	4.904	4.028–5.906	10.34	9.21
Singh et al. (19)	3,721	1.478	1.115–1.920	17.97	9.24
Corrêa et al. (20)	575	28.696	25.029–32.583	2.78	9.07
Singh et al. (21)	203	35.468	28.897–42.471	0.98	8.72
Total (fixed effects)	20,707	3.192	2.957–3.440	100.00	100.00
Total (random effects)	20,707	10.619	6.053–16.277	100.00	100.00
**Test for heterogeneity**					
Q	1,322.3511				
DF	10				
Significance level	*P* < 0.0001				
*I*^2^ (inconsistency)	99.24%				
95% CI for *I*^2^	99.07–99.38				
**Publication bias**					
Egger's test					
Intercept	20.1003				
95% CI	11.7959–28.4048				
Significance level	*P* = 0.0004				
Begg's test					
Kendall's Tau	0.6727				
Significance level	*P* = 0.0040				

**Table 7:** Pooled prevalence analysis of malaria in symptomatic pregnant women.

**References**	**Sample size**	**Proportion (%)**	**95% CI**	**Weight (%)**	
				**Fixed**	**Random**
Chauhan et al. (22)	120	12.500	7.168–19.778	4.95	18.35
Guin et al. (23)	500	7.400	5.264–10.056	20.48	20.66
Qureshi et al. (24)	1,222	10.720	9.041–12.591	50.00	21.17
Chawla and Manu (26)	416	6.490	4.320–9.303	17.05	20.50
Datta et al. (28)	183	34.973	28.085–42.356	7.52	19.33
Total (fixed effects)	2,441	10.737	9.538–12.032	100.00	100.00
Total (random effects)	2,441	13.131	7.192–20.519	100.00	100.00
**Test for heterogeneity**					
Q	82.2593				
DF	4				
Significance level	*P* < 0.0001				
*I*^2^ (inconsistency)	95.14%				
95% CI for *I*^2^	91.30–97.28				
**Publication bias**					
Egger's test					
Intercept	4.5794				
95% CI	−13.1941–22.3530				
Significance level	*P* = 0.4723				
Begg's test					
Kendall's Tau	0.2000				
Significance level	*P* = 0.6242				

**Figure 6:**
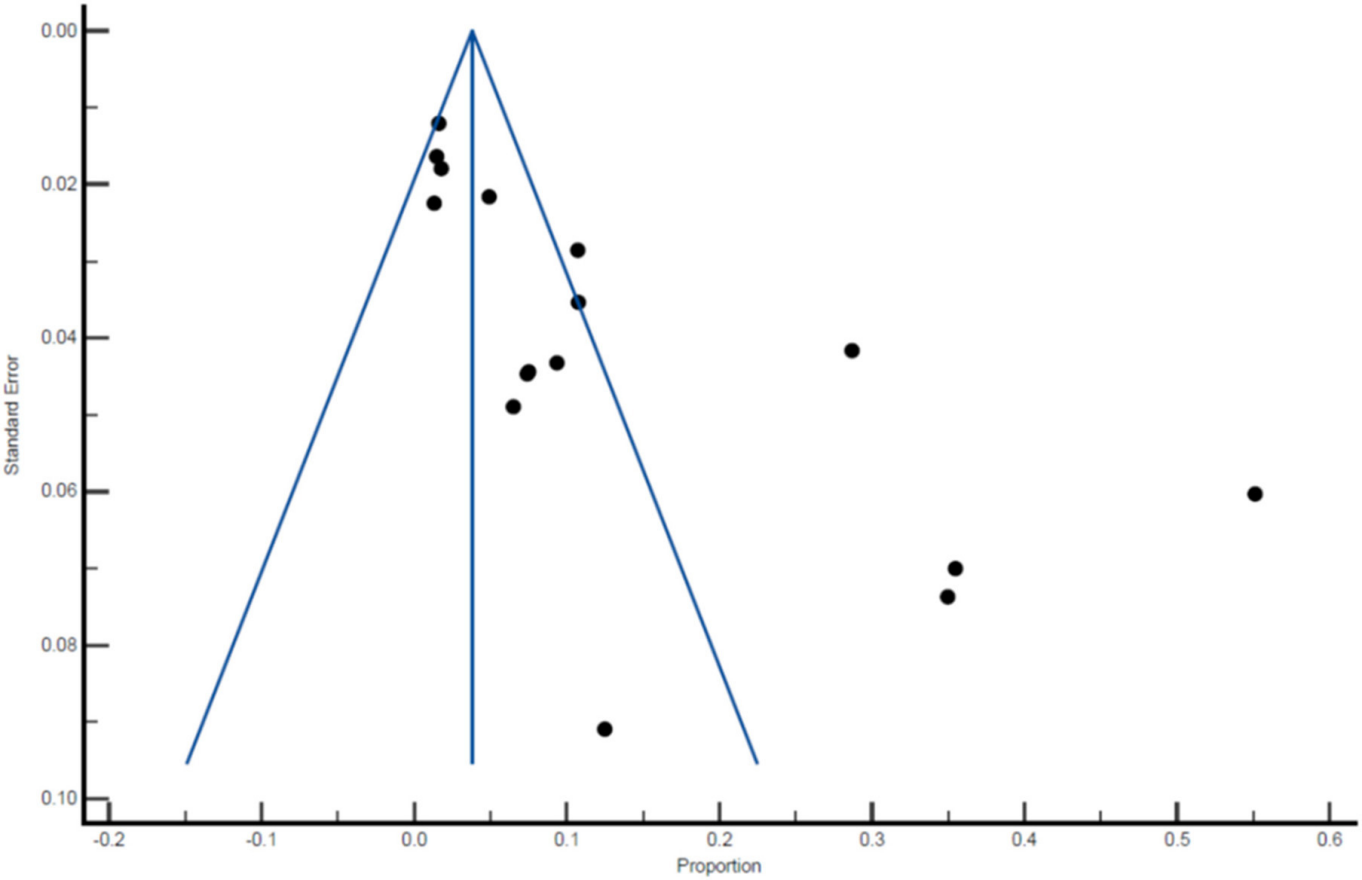
Funnel plot of the prevalence of malaria among pregnant women.

**Figure 7:**
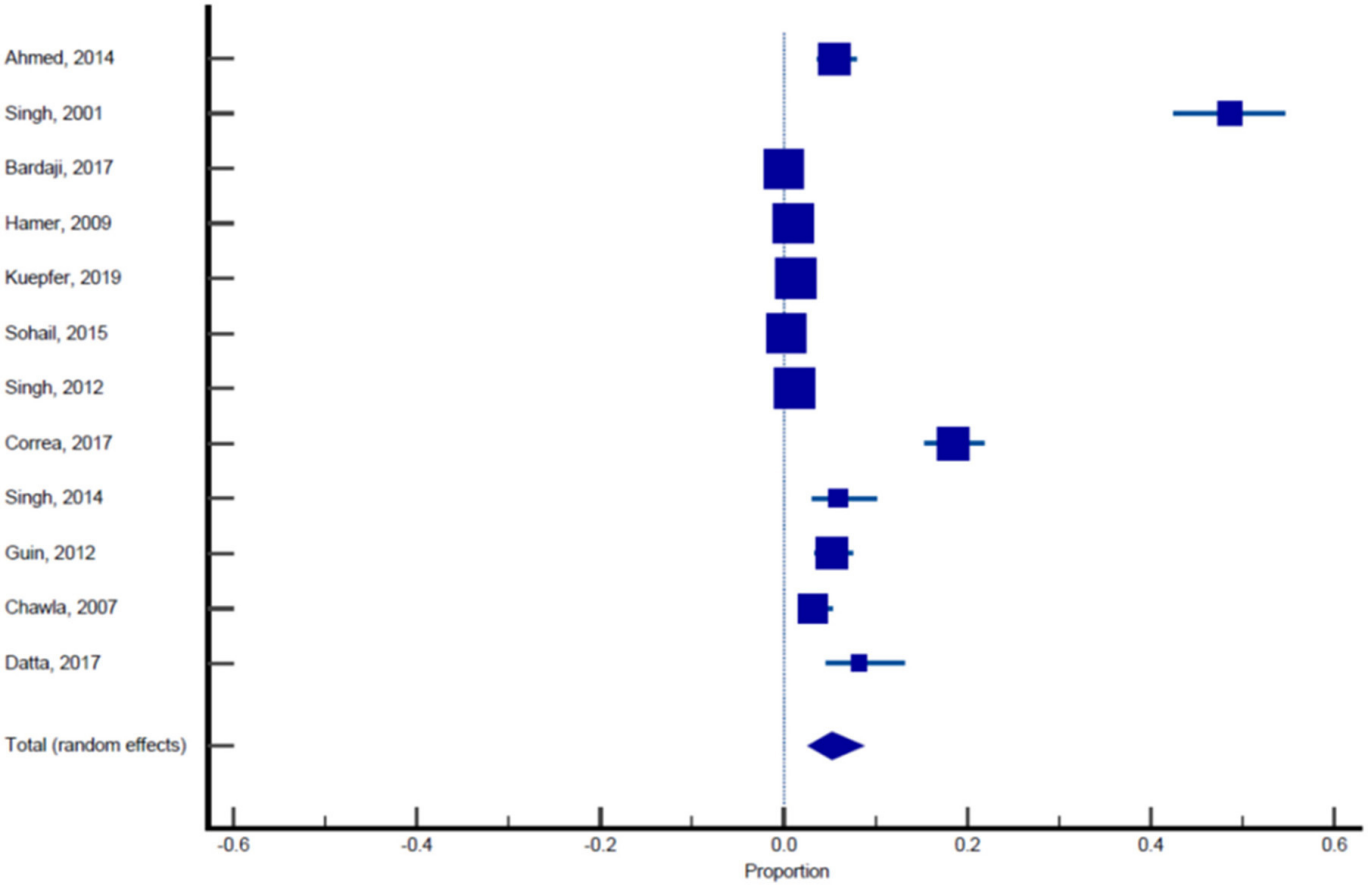
Pooled estimates of the prevalence of *Plasmodium falciparum* infection among pregnant women.

**Figure 8:**
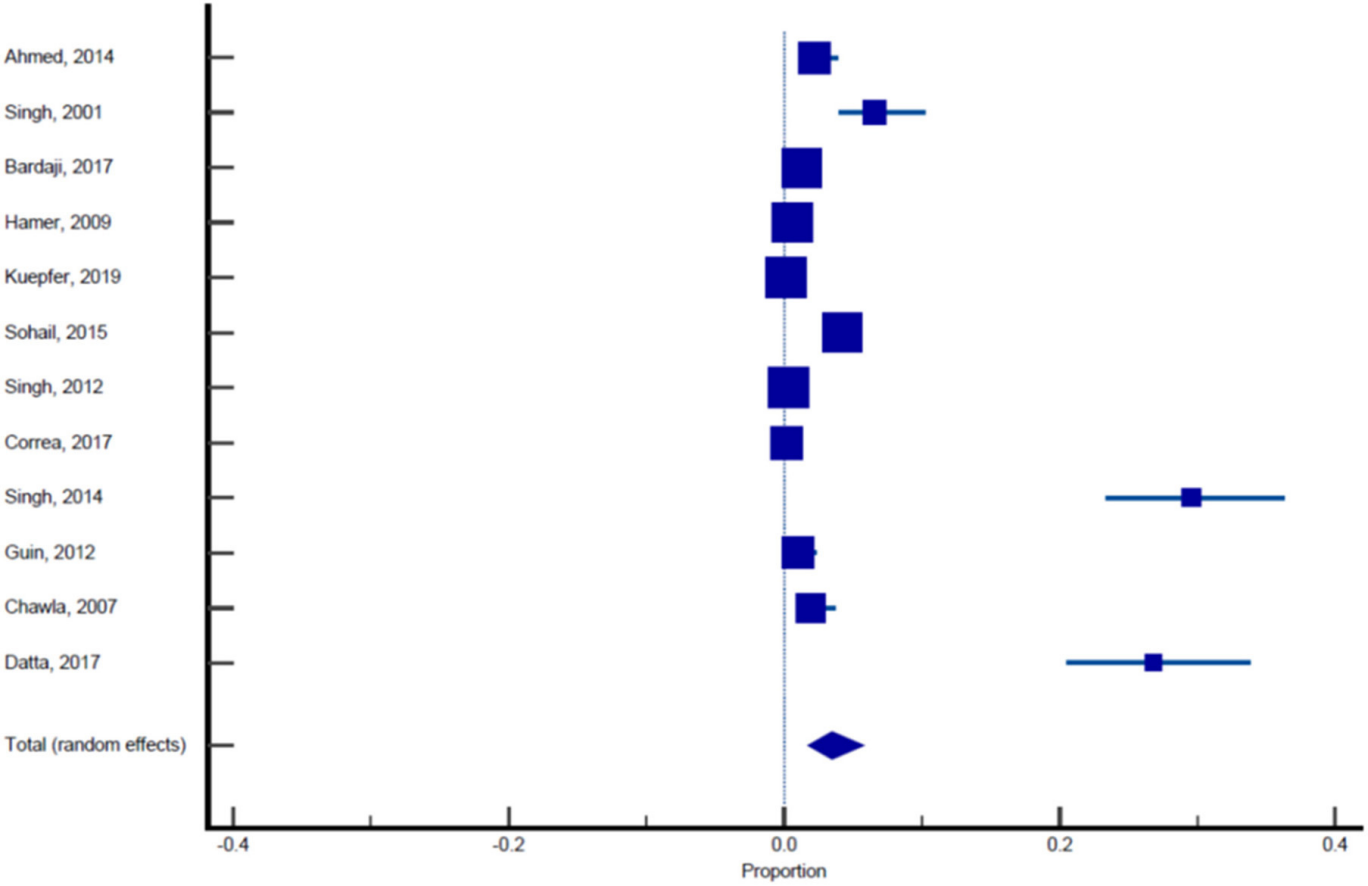
Pooled estimates of the prevalence of *Plasmodium vivax* infection among pregnant women.

**Figure 9:**
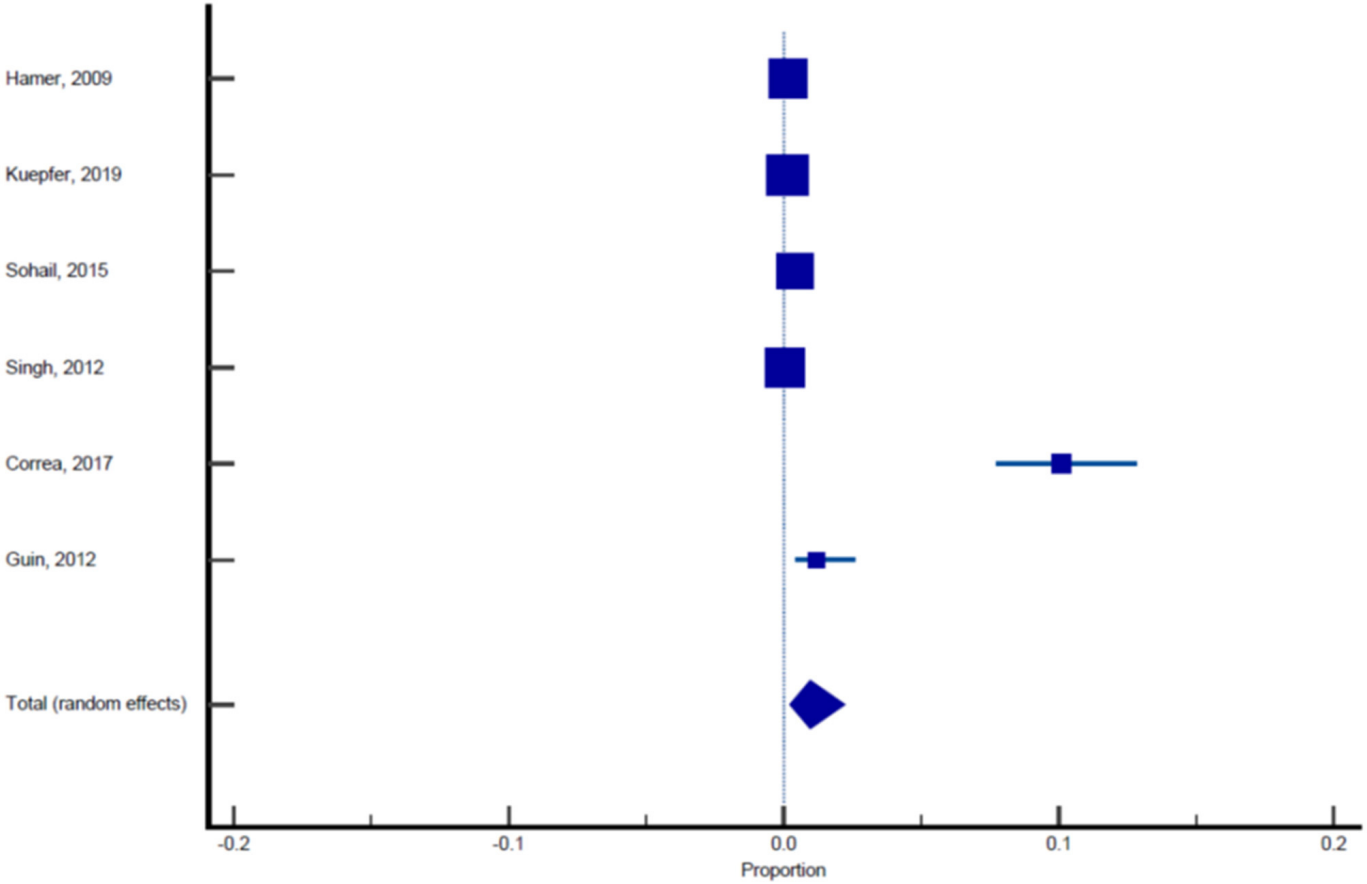
Pooled estimates of the prevalence of mixed infection among pregnant women.

There was an indication of publication bias across the included studies, as demonstrated by the asymmetrical distribution of the funnel plot (Figure 6). Among the 16 studies included in the final meta-analysis, 12 have reported the prevalence of *P. falciparum, P. vivax* or mixed infection within the same sample population. An overall pooled prevalence of different *Plasmodium* species was carried out and the prevalence was as follows: *P. falciparum* 5.25% (95% CI: 2.67, 8.66), *P. vivax* 3.46% (95% CI: 1.74, 5.75), and mixed infection 0.96% (95% CI: 0.22, 2.21; Tables 8–10, Figures 7–9).

**Table 8:** Pooled prevalence of *Plasmodium falciparum* infection in pregnant females.

**References**	**Sample size**	**Proportion (%)**	**95% CI**	**Weight (%)**	
				**Fixed**	**Random**
Ahmed et al. (11)	506	5.534	3.708–7.899	2.47	8.33
Singh et al. (12)	274	48.540	42.484–54.628	1.34	8.09
Bardaji et al. (14)	1,982	0.0505	0.00128–0.281	9.68	8.55
Hamer et al. (15)	3,104	1.031	0.706–1.452	15.16	8.58
Kuepfer et al. (16)	6,868	1.325	1.068–1.624	33.53	8.60
Sohail et al. (18)	2,141	0.234	0.0759–0.544	10.46	8.55
Singh et al. (19)	3,721	1.129	0.815–1.523	18.17	8.58
Corrêa et al. (20)	575	18.435	15.347–21.851	2.81	8.36
Singh et al. (21)	203	5.911	3.091–10.098	1.00	7.92
Guin et al. (23)	500	5.200	3.425–7.527	2.45	8.32
Chawla and Manu (26)	416	3.125	1.674–5.285	2.04	8.27
Datta et al. (28)	183	8.197	4.660–13.159	0.90	7.85
Total (fixed effects)	20,473	1.553	1.388–1.732	100.00	100.00
Total (random effects)	20,473	5.252	2.659–8.656	100.00	100.00
**Test for heterogeneity**					
Q	919.4662				
DF	11				
Significance level	*P* < 0.0001				
*I*^2^ (inconsistency)	98.80%				
95% CI for *I*^2^	98.50–99.05				
**Publication bias**					
Egger's test					
Intercept	10.9867				
95% CI	1.9327–20.0408				
Significance level	*P* = 0.0222				
Begg's test					
Kendall's Tau	0.2727				
Significance level	*P* = 0.2171				

**Table 9:** Pooled prevalence of *Plasmodium vivax* infection in pregnant females.

**References**	**Sample size**	**Proportion (%)**	**95% CI**	**Weight (%)**	
				**Fixed**	**Random**
Ahmed et al. (11)	506	2.174	1.090–3.856	2.47	8.32
Singh et al. (12)	274	6.569	3.940–10.184	1.34	7.97
Bardaji et al. (14)	1,982	1.261	0.818–1.856	9.68	8.65
Hamer et al. (15)	3,104	0.580	0.344–0.915	15.16	8.70
Kuepfer et al. (16)	6,868	0.160	0.0800–0.286	33.53	8.74
Sohail et al. (18)	2,141	4.250	3.436–5.193	10.46	8.66
Singh et al. (19)	3,721	0.296	0.148–0.528	18.17	8.71
Corrêa et al. (20)	575	0.174	0.00440–0.965	2.81	8.37
Singh et al. (21)	203	29.557	23.374–36.347	1.00	7.72
Guin et al. (23)	500	1.000	0.325–2.318	2.45	8.31
Chawla and Manu (26)	416	1.923	0.834–3.754	2.04	8.23
Datta et al. (28)	183	26.776	20.512–33.809	0.90	7.62
Total (fixed effects)	20,473	0.864	0.742–1.001	100.00	100.00
Total (random effects)	20,473	3.463	1.740–5.748	100.00	100.00
**Test for heterogeneity**					
Q	603.3980				
DF	11				
Significance level	*P* < 0.0001				
*I*^2^ (inconsistency)	98.18%				
95% CI for *I*^2^	97.64–98.59				
**Publication bias**					
Egger's test					
Intercept	10.0418				
95% CI	3.4797–16.6040				
Significance level	*P* = 0.0067				
Begg's test					
Kendall's Tau	0.6364				
Significance level	*P* = 0.0040				

**Table 10:** Pooled prevalence of mixed infection in pregnant females.

**References**	**Sample size**	**Proportion (%)**	**95% CI**	**Weight (%)**	
				**Fixed**	**Random**
Hamer et al. (15)	3,104	0.161	0.0523–0.376	18.36	17.16
Kuepfer et al. (16)	6,868	0.131	0.0599–0.249	40.61	17.35
Sohail et al. (18)	2,141	0.420	0.192–0.796	12.66	17.00
Singh et al. (19)	3,721	0.0537	0.00651–0.194	22.00	17.22
Corrêa et al. (20)	575	10.087	7.749–12.844	3.41	15.75
Guin et al. (23)	500	1.200	0.442–2.593	2.96	15.52
Total (fixed effects)	16,909	0.264	0.192–0.353	100.00	100.00
Total (random effects)	16,909	0.962	0.222–2.213	100.00	100.00
**Test for heterogeneity**					
Q	197.9010				
DF	5				
Significance level	*P* < 0.0001				
*I*^2^ (inconsistency)	97.47%				
95% CI for *I*^2^	96.13–98.35				
**Publication bias**					
Egger's test					
Intercept	11.3917				
95% CI	−0.9156–23.6989				
Significance level	*P* = 0.0620				
Begg's test					
Kendall's Tau	0.7333				
Significance level	*P* = 0.0388				

### Adverse Pregnancy Outcomes in Malaria Infected Pregnant Women

Narayanganj and Mandla district of Madhya Pradesh reported highest prevalence of 55% with 133 pregnant females testing positive for *P. falciparum* and 18 were tested positive for *P. vivax*. The same study also reported adverse pregnancy outcomes with six abortions and five still birth. Other districts have shown low prevalence with Bikaner district of Rajasthan reporting 1.3% prevalence. The report by Nayak et al. (30), from Bikaner district of Rajasthan, analyzed 25 pregnant women. They were tested microscopically, and found to be positive for *P. vivax*, along with comorbidity of anemia and thrombocytopenia that has resulted in eight preterm live birth, two intrauterine death, two stillbirth, two abortions, and 20 neonates with low birth weight. In another study carried out in the capital city of West Bengal by Datta et al. (28), 183 pregnant females were tested for malaria by microscopy and ELISA and 64 females came out to be positive with prevalence of 34%. Out of these 64 females, 15 were positive for *P. falciparum* and 49 for *P. vivax* contributing to 32 cases of preterm delivery, 51 cases of low birth weight, and 3 cases of perinatal mortality. Also, anemia and pathological placenta was found in 54 and 39 females respectively. Another case study carried in Vellore district of Tamil Nadu by Aleyamma et al. (29) where all the three females that were tested positive for *P. falciparum* by microscopy, had intrauterine fetal demise.

## Discussion

Out of 20 publications covered in this nationwide systematic review of malaria in pregnancy, there were 16 high quality studies that reported prevalence in asymptomatic or symptomatic pregnant women. Some of the highest prevalence were reported from the state of Madhya Pradesh which is one of the most endemic regions for malaria in India (11–13). Other states reported much lower prevalence. This wide range in prevalence can be due to several reasons. The primary reason, being the endemicity of malaria. Earlier studies have reported that five states, Jharkhand, Madhya Pradesh, Odisha, Uttar Pradesh, and Gujarat are responsible for 65% malaria cases reported from India (31). The population living in this area is at a much greater risk for developing the disease as compared to other northern, western and southern states of India. Other reasons are variable seasons in India, especially monsoon season which provide the suitable condition for mosquito breeding, poor medical infrastructure, lack of awareness among maternal and caregiver population about malaria and possibly poor immunity among them, might contribute toward high mortality and morbidity among pregnant women and their neonates (32–34).

Also, majority of the publications reported higher prevalence of *P. falciparum* than *P. vivax*, with only few publications showing higher prevalence of *P. vivax* (14, 18, 21, 23, 28) with almost no severe maternal or perinatal mortality and morbidity which could be proved by the fact that *P. vivax* is less dangerous for pregnant women as it does not attach to the epithelium of placenta causing lesser risk (35). Also, there were cases of extreme perinatal, neonatal and infant mortality and morbidity among population with higher *P. falciparum* infections. Other adverse outcomes were low birth weight, preterm births, abortions and still births. Other than this, overall, there were not many cases in which severe maternal outcomes were reported which could be due to moderate immunity among pregnant women in area of low transmission (36).

Apart from cross sectional studies, an analysis of hospital based observational studies, retrospective analysis and case series indicated malaria prevalence in symptomatic pregnant women. A study carried out by Nayak et al. (30) in the district of Bikaner reported 25 pregnant women, positive for *P. vivax* with extreme maternal effects such as severe anemia in 15 cases with 14 cases of preeclampsia and extreme perinatal outcome including eight preterm live birth, two intrauterine death, two still birth, two abortions, and 20 cases with low birth weight. Among all the cross-sectional studies reported in this review, only one publication by Guin et al. (23) reported in district of Jabalpur of Madhya Pradesh estimated maternal mortality with severe anemia and preeclampsia. This could be due to the socioeconomic status of Bhil's which is the largest tribal group of India condensed in Madhya Pradesh and the subject of this study. Delayed detection of disease among pregnant women resulting from poor accessibility to basic health infrastructure and poor awareness about prevention strategies and literacy rate might also contributes toward higher malaria prevalence.

Despite comprehensive search and analysis, several regions of India remained underrepresented in our systematic review either due to lack of comprehensive studies or due to lack of malaria prevalence, as India has a “National Framework for Malaria Elimination (NFME) in India 2016–2030,” that prioritizes malaria elimination by the year 2030. Many of these studies were carried out during different time frames which could lead to disparity in results. Most of the studies included women that choose to deliver in hospital and at least visited antenatal clinic during their pregnancy and by this it can be estimated that most of these women must have received preventive measures during their pregnancy such as chemoprophylaxis, which could further reduce the risk of extreme perinatal outcomes such as still births. We also observed publication bias based on the funnel plot. All 16 studies included in the final meta-analysis were not uniform in their study design as some were cross sectional studies while others were hospital based observational studies or retrospective analysis and may add to outcome variability. More in depth studies covering all aspects of malaria in pregnancy from other parts of India including northern, north-west region and south of India would have given a better understanding about prevalence of malaria in pregnancy. The health policy makers must include stringent preventive measures and improved access to malaria prevention, care and treatment to reduce the morbidity of malaria in pregnancy.

## Conclusion

Globally the burden of malaria in pregnancy is substantial with severe outcomes for maternal and neonatal health. Also, in endemic countries like India, it remains a major public health problem and contributes toward obstetric mortality, thus its prevention and control are a humanitarian priority. Assessment of its prevalence in India is crucial for targeting the at-risk population. Our systematic review estimates prevalence of pregnancy associated malaria and identifies geographical regions of high prevalence and associated adverse maternal and pregnancy outcomes. This information can be used by health agencies for targeted control program toward vulnerable and affected population. Our study also indicates that there is insufficient data for malaria in pregnancy from India, and more thorough and comprehensive analysis is required given the size and diversity of the country which will pave the way for improved maternal and neonatal health.

## Data Availability Statement

The original contributions presented in the study are included in the article/Supplementary Material, further inquiries can be directed to the corresponding author/s.

## Author Contributions

NS conceived the work. KJ and NS did the searches, extracted the data, and wrote the manuscript. PG, AB, and FD reviewed the extracted data. All authors reviewed and discussed the manuscript. All authors contributed to the article and approved the submitted version.

## Conflict of Interest

The authors declare that the research was conducted in the absence of any commercial or financial relationships that could be construed as a potential conflict of interest.

## Publisher's Note

All claims expressed in this article are solely those of the authors and do not necessarily represent those of their affiliated organizations, or those of the publisher, the editors and the reviewers. Any product that may be evaluated in this article, or claim that may be made by its manufacturer, is not guaranteed or endorsed by the publisher.

## Footnotes

^1^World Malaria Report. (2020).
