# Supplementary material for: Prevalence of Pregnancy Associated Malaria in India
Source: Front Glob Womens Health. 2022 May 26;3:832880. doi: 10.3389/fgwh.2022.832880 (PMC9178198; doi:10.3389/fgwh.2022.832880)
Supplement: Supplementary file 1 [file Table_1.DOCX]

Assessment of eligibility according to JBI scoring system

| Study | Was the sample frame appropriate to address the target population? | Were study participants sampled in an appropriate way? | Was the sample size adequate? | Were the study subjects and the setting described in detail? | Was the data analysis conducted with sufficient coverage of the identified sample? | Were valid methods used for the identification of the condition? | Was the condition measured in a standard, reliable way for all participants? | Was there appropriate statistical analysis? | Was the response rate adequate, and if not, was the low response rate managed appropriately? | Score |
| --- | --- | --- | --- | --- | --- | --- | --- | --- | --- | --- |
| Ahmed, 2014 | 1 | 1 | 1 | 1 | 1 | 1 | 1 | 1 | 1 | 9 |
| Singh, 2001 | 1 | 1 | 1 | 1 | 1 | 1 | 1 | 1 | 1 | 9 |
| Singh, 2005 | 1 | 1 | 1 | 1 | 1 | 1 | 1 | 1 | 1 | 9 |
| Bardaji, 2017 | 1 | 1 | 1 | 1 | 1 | 1 | 1 | 1 | 1 | 9 |
| Hamer, 2009 | 1 | 1 | 1 | 1 | 1 | 1 | 1 | 1 | 1 | 9 |
| Kuepfer, 2019 | 1 | 1 | 1 | 1 | 1 | 1 | 1 | 1 | 1 | 9 |
| Singh, 2018 | 1 | 1 | 1 | 1 | 1 | 1 | 1 | 1 | 1 | 9 |
| Sohail, 2015 | 1 | 1 | 1 | 1 | 1 | 1 | 1 | 1 | 1 | 9 |
| Singh, 2012 | 1 | 1 | 1 | 1 | 1 | 1 | 1 | 1 | 1 | 9 |
| Correa, 2017 | 1 | 1 | 1 | 1 | 1 | 1 | 1 | 1 | 1 | 9 |
| Singh, 2014 | 1 | 1 | 1 | 1 | 1 | 1 | 1 | 1 | 1 | 9 |
| Guin, 2012 | 1 | 1 | 1 | 1 | 1 | 1 | 1 | 1 | 1 | 9 |
| Chauhan, 2012 | 1 | 1 | 0 | 1 | 0 | 1 | 1 | 0 | 1 | 6 |
| Qureshi, 2014 | 0 | 0 | 1 | 0 | 1 | 1 | 1 | 0 | 1 | 5 |
| Munnur, 2005 | 0 | 0 | 1 | 0 | 1 | 0 | 0 | 1 | 1 | 4 |
| Chawla, 2007 | 0 | 1 | 1 | 0 | 0 | 1 | 1 | 0 | 1 | 5 |
| Bhadade, 2012 | 0 | 0 | 0 | 0 | 0 | 1 | 1 | 1 | 1 | 4 |
| Datta, 2017 | 1 | 1 | 0 | 0 | 0 | 1 | 1 | 1 | 1 | 6 |
|  |  |  |  |  |  |  |  |  |  |  |
